# Spontaneous Pneumomediastinum Associated with COVID-19 Pneumonia

**DOI:** 10.1155/2020/4969486

**Published:** 2020-10-20

**Authors:** Hamza Mimouni, Soufiane Diyas, Jamal Ouachaou, Ilyas Laaribi, Younes Oujidi, Manal Merbouh, Houssam Bkiyar, Brahim Housni

**Affiliations:** Department of Anesthesia and Intensive Care, Mohamed VI University Hospital Center, Mohamed I University, Oujda, Morocco

## Abstract

The first case of coronavirus disease 2019 (COVID-19) was declared in December in Wuhan, before becoming a global pandemic in a few weeks. Several complications of this infection have been reported. However, a spontaneous pneumomediastinum has rarely been described. We report the fourth case of this extremely rare complication in a 65-year-old male patient with severe acute respiratory syndrome coronavirus-2 (SARS-CoV-2) pneumonia, discovered during his therapeutic management by a CT scan control.

## 1. Introduction

COVID-19, originally reported in December 2019 in China, is currently spreading worldwide. It represents a major challenge for intensive care medicine, mainly due to the high number of intensive care admissions, the prolonged stay of patients, and the increased mortality in the presence of comorbidities.

Computed tomography of the chest has been proposed as triage and diagnostic tool for suspected COVID-19 patients. Although many parenchymal and extraparenchymal abnormalities have been observed, the presence of spontaneous pneumomediastinum remains a rarely described lesion.

We present a clinical case of a patient who tested positive for COVID-19 with spontaneous pneumomediastinum.

## 2. Case Report

We report the case of a 65-year-old patient who was admitted to intensive care for the management of respiratory distress and who has no medical or surgical history, with no history of cigarette smoking and not under any treatment.

His history reveals an influenza-like illness, a fever at 39.2 °C, arthralgia, myalgia associated with a dry cough, and anosmia with the notion of contact with a positive COVID-19 patient.

On admission, clinical examination found a conscious, tachypneic patient at 24 cycles/min with O_2_ saturation at 88% in ambient air and 93% under 2 L/min of oxygen at the telescope, a heart rate of 82 beats per minute, and blood pressure at 120/80 mmHg. Moreover, the examination confirmed regular heart sounds and no audible breath, and there was no sign of right or left heart failure. Pulmonary auscultation found high-sounding gasps and sibilant in bilateral.

The blood gas analysis revealed a pH of 7.3, PaO_2_ of 53 mmHg, PaCO_2_ of 46 mmHg, and HCO_3_^−^ of 22 mmol/L^−1^.

The biological assessment found an aspecific inflammatory syndrome, with lymphopenia at 2400 cells/*μ*L, C-reactive protein at 110 mg/L, and procalcitonin at 0.17 ng/ml, a normal hemoglobin rate at 14.8 g/dL, a normal kidney function with urea at 5.66 mmol/L, and creatinine at 8 *μ*mol/L.

**Figure 1:**
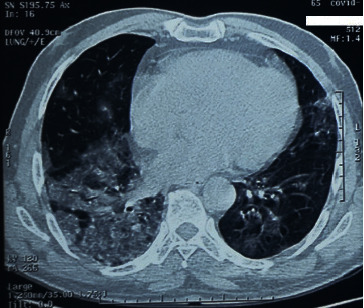
Chest CT scan in the axial section of the mediastinal window showing ground glass images with an asymmetrical peripheral arrangement under the pleural layer, producing a crazy-paving appearance characteristic of typical COVID-19 pneumonia.

A chest CT scan was carried out objectifying bilateral, peripheral, and subpleural ground-glass opacities, with foci of band condensation (Figure 1), suggestive of an infection by SARS-CoV-2 confirmed by polymerase chain reaction (PCR).

Our patient gradually presented with dyspnea at rest with signs of struggle associated with moderate to severe basal thoracic pain, requiring oxygen therapy with a high concentration facial mask reaching up to 12 L/min. Front chest radiographs showed a stationary aspect of the lesions with bilateral reticulonodular opacities especially basal and more marked on the right side in connection with an interstitial syndrome.

**Figure 2:**
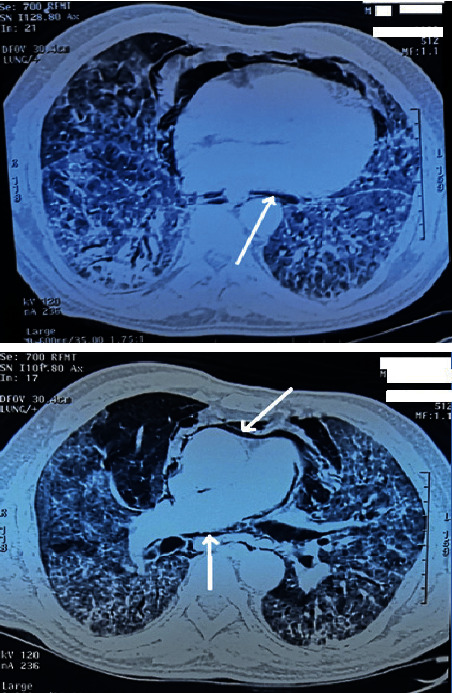
Chest CT scan in the axial section of the pulmonary parenchymal window showing a low-to-medium abundant pneumomediastinum (arrow) dissecting the large mediastinal vessels in a 65-year-old patient with COVID-19 pneumonia.

Faced with the nonimprovement of the clinical picture after a therapeutic protocol associating hydroxychloroquine and azithromycin, the patient received an immunoglobulin treatment at a rate of 1 g/kg for 5 days and then administration of two doses of tocilizumab 400 mg at 48 h interval. On the 16th day, a computed tomography of the chest was performed for a better study of the parenchyma (millimeter sections in high resolution with an injection of contrast product) revealing a spontaneous pneumomediastinum with no sign in favor of subcutaneous emphysema or pulmonary embolism (Figure 2).

The management of this rare complication was monitoring of respiratory rate, oxygen saturation, heart rate, and blood pressure with analgesic treatment for chest pain.

The clinical course is favorable with a partial improvement in respiratory symptoms, and our patient is declared cured after 31 days and 2 negative PCR on nasopharyngeal swabs taken 24 hours apart.

## 3. Discussion

Since December 2019, a series of cases of viral pneumonia caused by a new coronavirus identified on samples of the airways named SARS-CoV-2 appeared in Wuhan in China and quickly propagated all over the world.

The main clinical signs during SARS-CoV-2 infection described in three studies by Guan et al. were associated with a fever higher than 37.5°C, a cough, sputum, and dyspnea, occurring in the first day of infection [1]. Also, myalgia was frequent, and digestive signs, which may be inaugural events, were also reported, such as diarrhea and nausea/vomiting.

The chest CT plays a pivotal role in the triage of patients arriving in the emergency room, and the most frequent characteristic CT abnormalities of COVID-19 pneumonia are areas of ground glass, multifocal, bilateral, and asymmetrical. The involvement classically predominates in the peripheral, posterior, and basal regions. Other atypical CT signs appear in the form of pseudonodular condensations, sometimes accompanied by an inverted halo sign [2, 3].

Spontaneous pneumomediastinum is rare in SARS-CoV-2 lung infection. Only 3 cases were reported to date [4–6]. It is characterized by the presence of air in the mediastinal structures with no apparent cause (trauma and iatrogenicity).

Physiopathologically a spontaneous pneumomediastinum is secondary to an endobronchial hyperpressure (effort with closed glottis) causing an alveolar rupture and then gas dissemination in the peri-broncho-vascular space up to the pulmonary hilum and the mediastinum [7].

The most common symptom of spontaneous pneumomediastinum is acute retrosternal chest pain, followed by dyspnea, while subcutaneous emphysema is not systematic. Treatment is symptomatic, and no specific treatment seems necessary. Oxygen therapy is not systematic although it would accelerate the resorption of pneumomediastinum (absorption of free air by increasing nitrogen concentrations) [8, 9]. In the case of lung infections due to SARS-CoV-2, the virus causes the degradation of the integrity of the alveolar membrane because it infects type II pneumocytes. Consequently, the damage of the alveolar membrane due to SARS-CoV-2 infection may be one of the mechanisms leading to alveolar rupture and the appearance of spontaneous pneumomediastinum [10].

Our case represents the 4th case described in the literature and the 2nd case described in Morocco. Two hypotheses could explain the mechanism of pneumomediastinum in our case: firstly, the coughing effort, and secondly, the spontaneous rupture of the alveolar partitions secondary to the alveolar and interstitial lesions induced by SARS-CoV-2 infection.

## 4. Conclusion

Spontaneous pneumomediastinum remains a presentation rarely encountered in the diagnosis and monitoring of patients with COVID-19 and can potentially be an aggravating factor in the prognosis of patients with COVID-19 pneumonia. Indeed, the association of a pneumomediastinum and a parenchymal lesion extended on the thoracic scanner witnessing significant destruction of the alveolar membrane. However, the occurrence of pneumomediastinum in a patient hospitalized for COVID-19 pneumonia requires close monitoring of the patient.

## Data Availability

The data used to support the findings of this study are available from the corresponding author upon request.
